# The effects of concurrent training on physical fitness in children and adolescents: a systematic review and meta-analysis

**DOI:** 10.3389/fped.2026.1800025

**Published:** 2026-04-10

**Authors:** Fei Cui, Zhuojing Sun, Mingchen Ma, Anqi Dong, Chen Xu, Jiaju Zhu, Guodong Ma

**Affiliations:** 1 Graduate School, Jilin Sport University, Changchun, Jilin, China; 2 School of Physical Education, Northeast Normal University, Changchun, Jilin, China; 3 Human Movement Science College, Jilin Sport University, Changchun, Jilin, China

**Keywords:** children and adolescents, concurrent training, endurance training, physical fitness, strength training

## Abstract

**Objective:**

To use a meta-analysis to explore the effects of concurrent training on physical fitness in a population aged (10∼24 years), and to further analyze differences related to factors such as intervention sequence, duration, and frequency.

**Method:**

The search platform includes data resource systems such as PubMed, Web of Science, Scopus, CNKI, and VIP, with inclusion and exclusion criteria established based on PICOS. Review Manager 5.4 was used for quality assessment and statistical analysis.

**Results:**

Compared to isolated strength training or endurance training, combined training significantly enhances lower-body explosive power in children and adolescents (MD = 0.05, 95% CI: [0.01, 0.09], I^2^= 0%, *P* = 0.009) and aerobic capacity (MD = 2.05, 95% CI: [0.80, 3.31], I^2^= 82%, *P* = 0.001). Subgroup analysis revealed that training strength before endurance within the same period (MD = 2.06, 95% CI: [0.73, 3.39], I^2^= 83%, *P* = 0.002) and the absence of an interval between the two training sessions (MD = 2.09, 95% CI: [0.50, 3.68], I^2^= 83%, *P* = 0.01) were associated with greater improvements in VO₂ max in the pediatric and adolescent population. An 8-week concurrent training intervention period resulted in improvements in both maximal oxygen uptake and lower-body explosive power.

**Conclusion:**

Concurrent training ≤3 times per week does not produce “interference effects” in children and adolescents; concurrent training can significantly improve cardiorespiratory endurance while maintaining strength levels in this population.

**Systematic Review Registration:**

https://www.crd.york.ac.uk/PROSPERO/view/CRD420251242860, PROSPERO CRD420251242860.

## 1 Introduction

Healthy growth and development during childhood and adolescence are influenced by social, nutritional, and environmental factors within the family, school, and community (1). Promoting health can help address deficiencies in children and adolescents during their developmental stages; therefore, prioritizing their health is crucial for a smooth transition into adulthood (2, 3).The World Health Organisation recommended that children and adolescents engage in an average of 60 minutes of moderate to vigorous aerobic physical activity daily throughout the week. This not only enhances physical fitness but also reduces the incidence of chronic conditions such as obesity (4, 5). Both underweight and obesity are detrimental to overall health throughout life; maintaining a healthy weight is essential for sustaining well-being (6). A meta-analysis of 298 population-based surveys involving 1.6 million participants revealed that 81% of children and adolescents aged 11–17 years failed to meet the World Health Organization's physical activity guidelines, thereby being classified as physically inactive (7, 8). This is consistent with the findings of other studies, including those with large sample sizes (9–11). This reflects the global trend of insufficient physical activity and sedentary behavior among children and adolescents (12). It is evident that the physical health levels of children and adolescents need improvement.

Cardiorespiratory fitness (CRF) is a key factor influencing the levels of physical activity and fundamental motor skills in children and adolescents (13). Endurance training (ET) can improve cardiopulmonary function, enhance cardiopulmonary endurance, and increase resistance to fatigue (14). Muscular strength (MS) is one of the core elements for the healthy growth of children and adolescents (15). Strength training can improve muscle strength in children and adolescents and enhance explosive power (16, 17). However, a single training mode has limited effectiveness in improving functional capacity (18). Concurrent training (CT) refers to a training method where strength and endurance training tasks are scheduled during the same period. The earliest research on this topic appeared in a 1980 study by American exercise physiologist Hickson. This research revealed that concurrent training in aerobic and strength disciplines does not simply yield a cumulative effect of strength and endurance gains, but rather involves a degree of “internal dissipation" (19). Research indicates that concurrent strength and endurance training may interfere with each other (20). This leads to diminished strength training results and the emergence of an “interference effect" (19, 21). However, some researchers have not observed the “interference effect” occurring in similar studies (22, 23).

Most evidence regarding CT interference effects originates from adult and animal studies. Given physiological and biomechanical differences between pediatric and adolescent populations and middle-aged/elderly individuals, the response to CT in pediatric and adolescent groups may be biased (18, 24). Given the significant importance of CT in enhancing the physical fitness of children and adolescents, as well as existing controversies surrounding it, this paper employ meta-analysis to synthesize existing research findings. It objectively evaluates the impact of CT on the physical fitness of this demographic and provides a reference for selecting optimal CT training programs.It can provide a theoretical basis for physical education instruction in schools and contribute to the healthy and sustainable development of adolescents.

## 2 Research methods

This research protocol has been registered with the international prospective systematic review registry platform PROSPERO, registration number: CRD420251242860.

### 2.1 Literature search strategy

The relevant literature cited in this paper was primarily sourced from five databases: PubMed, Web of Science, Scopus, China National Knowledge Infrastructure (CNKI), and VIP. The search covered the period from the inception of each database through November 2025. Additionally, a back-search method was employed to supplement the retrieved literature, with the final search conducted on November 20, 2025. Chinese keywords include: “concurrent training,” “concurrent strength and endurance training,” “concurrent resistance and endurance training,” “children,” “adolescents,” etc. English topics include: “concurrent training”、“concurrent strength and endurance training”、“combined strength and endurance training”、“combined resistance and endurance training”、“simultaneous training”、“teenager”、“adolescent”、“youth” and their combinations. Table 1 details the specific steps of the PubMed search process.

**Table 1:** Pubmed database search method.

Search procedure	Search query
#1	“concurrent training”[Title/Abstract] OR “concurrent strength and endurance training”[Title/Abstract] OR “combined strength and endurance training” [Title/Abstract] OR “combined resistance and endurance training” [Title/Abstract] OR “simultaneous training” [Title/Abstract]
#2	“Teenager” [Title/Abstract] OR “adolescent”[Title/Abstract] OR “youth” [Title/Abstract] OR “youngster” [Title/Abstract] OR “child” [Title/Abstract] OR “puberty” [Title/Abstract]
#3	#1 AND #2

### 2.2 Inclusion and exclusion criteria for literature

This paper was strictly written in accordance with the PRISMA statement for systematic reviews and meta-analyses, while the inclusion and exclusion criteria for literature were established based on the PICOS principles. The inclusion criteria for this study were as follows: ① Study participants were healthy individuals aged 10–24 years old, falling within the age range of 0–24 years defined by the WHO 2020 Manual for children and adolescents. ② Intervention measures consist of concurrent training with an intervention period of ≥8 weeks; ③ The control group comprised strength training, endurance training, and no training program; ④Outcome measures include one of the following: BMI, vertical jump, standing long jump, medicine ball throw (1-kg), medicine ball throw (3-kg), 20-meter sprint, VO₂max, body fat percentage, or maximum lower-body strength; ⑤ Experimental research.

Exclusion Criteria: ① Animal studies; ② Unable to obtain full-text articles or extract valid data; ③ Control group: non-pure strength and endurance training; ④ Review papers, conference papers; ⑤ Research content inconsistencies; ⑥ Outcome measures inconsistent with the literature.

### 2.3 Literature screening and data extraction

Using EndNote 20 to remove duplicate references, followed by screening out irrelevant references based on titles and abstracts, and then reviewing the full texts according to the inclusion and exclusion criteria to remove those that do not meet the requirements. This process was conducted independently by two researchers; in the event of a disagreement, a decision was reached after discussion with a third researcher.

Data extraction: ① First author; ② Publication year; ③ Age, number, and grouping; ④ Training characteristics; ⑤ Outcome measures.

### 2.4 Bias risk assessment

The “Risk of Bias Assessment” tool from the Cochrane Collaboration was used, which includes: (1) whether randomization was performed; (2) concealment of the allocation plan; (3) whether participants and study personnel were blinded; (4) blinding of outcome assessment; (5) completeness of reporting; (6) selective reporting of study results; and (7) the presence of other sources of bias (25).

### 2.5 Statistical methods

Forest plots and funnel plots were generated using RevMan 5.4 software, and subgroup analyses were conducted. Since all relevant indicators included in this study were continuous variables with inconsistent measurement methods and units, the standardized mean difference (SMD) and its 95% confidence interval (CI) were used as measures of effect. If the 95% CI included 0, it indicated that the comparison between the two interventions was not statistically significant. Additionally, the I^2^ value was used as an indicator of heterogeneity; if *P* > 0.1 and and I^2^ < 50%, the studies were considered homogeneous, and a fixed-effects model was adopted. Conversely (*P* ≤ 0.1, I^2^ ≥ 50%), a random-effects model was used, and sensitivity analyses were conducted to assess the stability of the results. Subsequently, subgroup analyses were performed to explore the sources of heterogeneity, primarily focusing on gender differences and intervention protocols (intervention duration and frequency) as moderator variables. A funnel plot was used to assess whether publication bias was present in the included studies. Sensitivity analysis was performed using RStudio version 2023.09.1 + 494 (R software version 4.4.1) with the metafor package (version 4.6-0), and Egger's test was used to assess publication bias.

## 3 Result

### 3.1 Literature screening results

**Figure 1:**
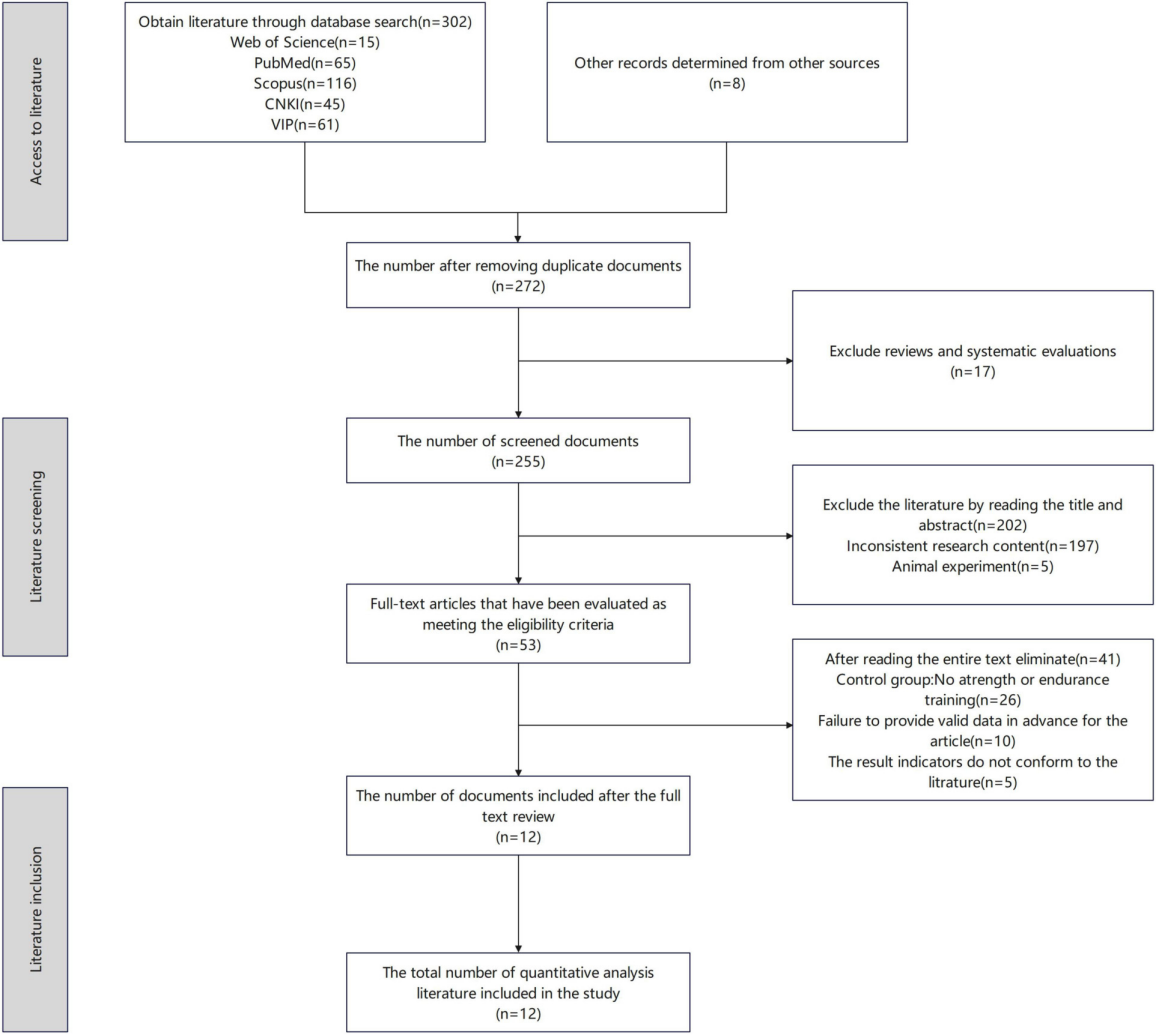
Literature inclusion flowchart.

A total of 302 articles were identified in this study, with eight additional articles identified through back-searching. After deduplication, 272 articles remained; after excluding irrelevant studies, 53 articles remained.Following a full-text review, 12 articles were included in the analysis, comprising four Chinese articles (26–29), and eight English articles (30–37),as shown in Figure 1.

### 3.2 Basic information of included literature

This study included 12 publications comprising 30 research studies (one study extracted from (32), two studies each from (26, 27, 30, 33–36), three studies from (37), and four studies from (28, 29, 31). Sample size: 781 participants, with 389 in the experimental group and 392 in the control group. Detailed information is provided in Table 2.

**Table 2:** Basic experimental characteristics of the literature included in this study.

First Author (Year)	Grouping	Age	Intervention			Training Content	Outcome Indicator
			Week	Frequency（t/w）	Intensity		
SHAWN P. GLOWACKI (35)（2004）	CT = 16	22 ± 2	12	2	3 × 10Second75%1RM∼3 × 6Second85%1RM、20min65%HRmax∼40min80%HRmax	RA 、TD	②⑦⑧⑨
	ST = 13	23 ± 3			3 × 10Second75%1RM∼3 × 6Second85%1RM	RA	
JEFFREY C. GERGLEY (36)（2009）	CT1 = 10	20.30 ± 1.56	9	2	3 × 12RM∼3 × 8RM、20min65%HRmax∼40min65%HRmax	RA、PB	⑨
	CT2 = 10	19.70 ± 1.56			3 × 12RM∼3 × 8RM、20min65%HRmax∼40min65%HRmax	RA、PB	
	ST = 10	20.70 ± 1.49			3 × 12RM∼3 × 8RM	RA	
Albano Santos (33)（2011）	CT = 25	13.5 ± 1.03	8	2	75%VO2max	T、Z、C、P	①②③④⑤⑥⑦⑧
	ST = 21				—	RA	
	GT = 21				—	—	
ALBANO P. SANTOS (34) （2012）	CT = 15	13.3 ± 1.04	8	2	75%VO2max	T、Z、C、P	①②③④⑤⑥
	ST = 15				—	RA	
	GT = 12				—	—	
Mohammad Hassan Ferdosi (37)（2012）	CT = 12	21.38 ± 2.06	8	3	25min65%HRmax∼40min85%HRmax	P、RA	①⑦
	ST = 12	21.0 ± 1.57			—	RA	
	ET = 12	22.0 ± 0.89			25min65%HRmax∼40min85%HRmax	P	
	GT = 12	21.44 ± 1.13			—	—	
ANA R. ALVES (31)（2016）	CT1 = 45	10.9 ± 0.5	8	2	75%VO2max	T、Z、C、P	②③④⑤⑥⑦
	CT2 = 38				75%VO2max	T、Z、C、P	
	ST = 41				—	RA	
	GT = 44				—	—	
Marwa Bouamra (32)（2022）	CT = 13	13.2 ± 0.9	9	3	3 × 6Second75%1RM	C、RA	①②⑧
	ST = 12	12.7 ± 0.9			3 × 6Second75%1RM	RA	
Lin Jianjian (27)（2024）	CT1 = 20	10.49 ± 1.92	12	3	70∼80% VO2max	Z、C、P	⑧
	CT2 = 20	10.18 ± 1.66			70∼80% VO2max	Z、C、P	
	ST = 20	10.06 ± 2.71			—	—	
Wu Jihao (28)（2024a）	CT1 = 10	18.91 ± 0.77	8	2	1RM70% 60—70% VO2max	RA、PB	⑦⑨
	CT2 = 10	18.79 ± 0.71			1RM70% 60—70% VO2max	RA、PB	
	ST = 10	18.95 ± 0.73			1RM70%	RA	
	ET = 10	18.83 ± 0.81			60—70% VO2max	PB	
Wu Jihao (29)（2024b）	CT1 = 17	18.85 ± 0.73	12	2	3 × 6Second75%1RM 30min50%—80%HRmax	RA、PB	⑦⑨
	CT2 = 17	18.81 ± 0.71			3 × 6Second75%1RM 30min50%—80%HRmax	RA、PB	
	ST = 16	18.91 ± 0.77			3 × 6Second75%1RM	RA	
	ET = 16	18.89 ± 0.81			30min50%—80%HRmax	PB	
Zhen Li (30)（2024）	CT1 = 50	12.6 ± 0.3	12	2	8-20Second1RM 50%-85%HRmax	P、RA	③⑦
	CT2 = 50	12.5 ± 0.4			8-20Second1RM 50%-85%HRmax	P、RA	
	GC = 50	12.4 ± 0.3			—	—	
Shi Shuyun (26)（2025）	CT = 15	19.85 ± 0.90	8	2	3 × 6Second70%—80%1RM 30min60%—75%HRmax	RA、HB	⑦⑨
	ST = 14	19.71 ± 1.25			3 × 6Second70%—80%1RM	RA	
	ET = 15	18.57 ± 0.53			30min60%—75%HRmax	HB	

CT denotes concurrent training, ST denotes strength training, ET denotes endurance training, GT denotes no training program. Training content: T denotes throwing; Z denotes vertical jump box; C denotes sprinting; P denotes running; RA denotes resistance equipment; HB denotes Hand-Crank Exercise Bike Trainer; PB denotes Power Bike; TD denotes treadmills; "—” indicates unspecified. Outcome Measures: ① Body Mass Index (BMI) ② Vertical Jump Height ③ Standing Long Jump ④ Medicine Ball Throw (1-kg) ⑤ Medicine Ball Throw (3-kg) ⑥ 20-M Sprint ⑦ VO₂ max ⑧ Body Fat Percentage ⑨ Lower-Body 1RM Strength.

### 3.3 Inclusion in literature quality assessment

**Figure 2:**
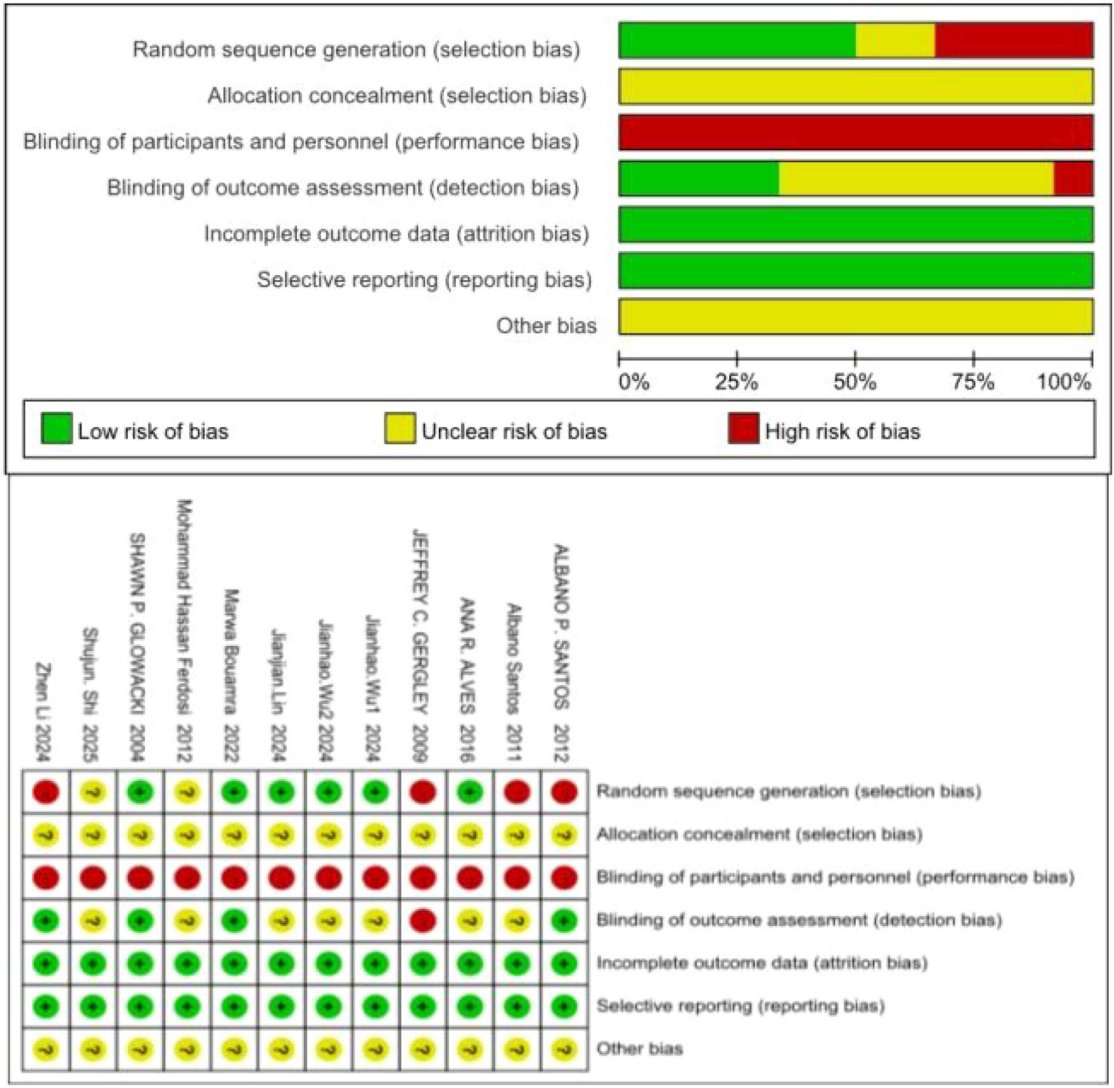
Risk of bias plot for included studies.

The Cochrane Collaboration's“Risk of Bias Assessment”indicates that, regarding the assessment of risk of bias in the generation of random sequences, four studies were classified as high risk because they did not provide detailed descriptions of the methods used; two studies were classified as uncertain risk because they lacked specific methodological descriptions; and 6 studies were classified as low risk. Regarding the risk of allocation concealment, the 12 included studies were classified as having an uncertain risk because the articles did not mention whether allocation concealment measures were implemented; regarding the risk of participant and staff blinding, all 12 included studies were classified as high risk; however, since this study involved an exercise intervention, the lack of blinding for participants did not affect the experiment; Regarding the risk of blinding in outcome assessment, only one study did not specify whether the outcome assessors were blinded to group assignments, posing a high risk. In seven studies, some tests were objectively measured, resulting in an uncertain risk. Four studies reported that all outcome measures were objectively measured and subject to minimal subjective influence, posing a low risk; regarding incomplete outcome data, all 12 included studies had good data completeness, posing a low risk; Regarding selective reporting, all 12 included studies reported the relevant indicators, indicating a low risk; regarding other risks of bias, the risk status for all 12 included studies was uncertain (see Figure 2).

### 3.4 Meta-analysis results

#### 3.4.1 BMI

**Figure 3:**
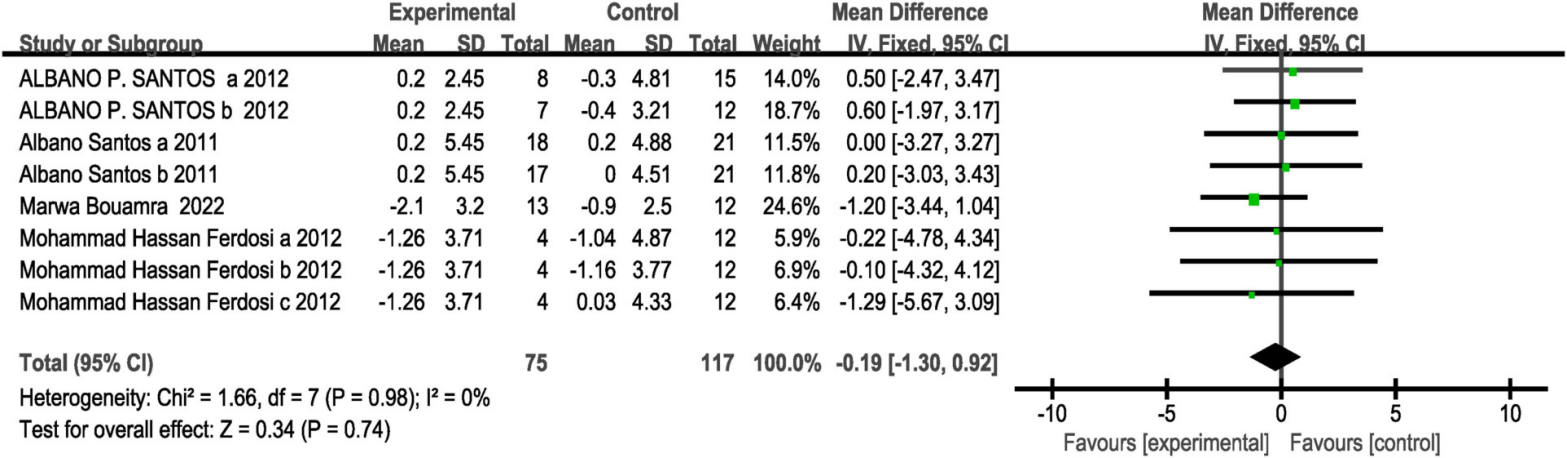
Forest plot of the effect of concurrent training on BMI.

A total of four studies (eight research projects, with 75 participants in the experimental group and 117 in the control group) were included regarding the BMI index. As there was no heterogeneity in the results (I^2^= 0%, *P* > 0.1), a fixed-effects model was selected for analysis. The results showed no significant difference between the two groups [MD = −0.19, 95% CI: (−1.30, 0.92), *P* = 0.75 > 0.05], indicating that the effect was not statistically significant. This suggests that CT training did not have a significant impact on body mass compared to the control group, as shown in Figure 3.

Subgroup analysis results showed that there was no statistically significant difference in the effect on body mass index (BMI) between intervention frequencies of two times per week [MD = 0.37, 95% CI: (−1.12, 1.85), I^2^ = 0%, *P* = 0.63 > 0.05] and three times per week (MD = −0.91, 95% CI: [−2.58, 0.77], I^2^ = 0%, *P* = 0.29 > 0.05) showed no statistically significant difference in their effects on body mass index (BMI) (*P* > 0.05 between subgroups). All four studies included for this outcome measure had an eight-week intervention period, with CT training following the sequence of strength training followed by endurance training, and no rest period between strength and endurance training. Therefore, subgroup analysis was performed only for the intervention frequency variable; see Table 3.

**Table 3:** Subgroup analysis results for each experimental outcome measure in the included studies.

Outcome Indicator	Variable	Category	Number of documents	Effect Model	MD	95%CI	I^2^ （%）	Intergroup Heterogeneity Test Q df P		
BMI	Intervention frequency	Twice a week	2	FE	0.37	[−1.12,1.85]	0	1.24	1	0.26
		3 times/week	2		−0.91	[−2.58,0.77]	0			
CMJ	Intervention interval	Interval	1	FE	1.65	[−0.86,4.16]	0	1.64	1	0.20
		Immediately	5		0.01	[−0.01,0.03]	31			
	Intervention Cycle	8W	3	FE	0.01	[−0.01,0.03]	27	0.33	1	0.57
		12W	1		−2.00	[−8.91,4.91]	NA			
	Intervention frequency	Twice a week	4	FE	0.01	[−0.01,0.03]	19	1.99	1	0.16
		3 times/week	1		−2.60	[−6.23,1.03]	NA			
SLJ	Intervention interval	Interval	2	FE	**0** **.** **07**	**[0.01,0.13]**	0	0.50	1	0.48
		Immediately	4		0.04	[−0.01,0.09]	0			
	Intervention Cycle	8W	3	FE	**0**.**05**	**[0.00,0.10]**	0	0.00	1	1.00
		12W	1		0.05	[−0.01,0.11]	0			
1-kg solid ball	Intervention interval	Interval	1	FE	0.10	[−0.16,0.36]	0	0.22	1	0.64
		Immediately	3		0.18	[−0.02,0.37]	28			
3-kg solid ball	Intervention interval	Interval	1	FE	0.06	[−0.11,0.24]	0	0.13	1	0.72
		Immediately	3		0.10	[−0.03,0.24]	43			
20-meter sprint	Intervention interval	Interval	1	FE	−0.05	[−0.19,0.09]	0	0.00	1	0.95
		Immediately	3		−0.05	[−0.14,0.04]	6			
VO_2_max	Intervention sequence	ST + ET	8	RE	**2**.**06**	**[0.73,3.39]**	83	0.00	1	0.96
		ET + ST	1		2.15	[−1.59,5.89]	42			
	Intervention interval	Interval	3	RE	1.99	[−0.11,4.09]	82	0.01	1	0.94
		Immediately	9		**2**.**09**	**[0.50,3.68]**	83			
	Intervention Cycle	8W	6	RE	**2**.**10**	**[0.61,3.59]**	81	0.01	1	0.91
		12W	3		1.93	[−0.65,4.52]	85			
	Intervention frequency	Twice a week	8	RE	**1**.**93**	**[0.61,3.25]**	84	0.60	1	0.44
		3 times/week	1		3.64	[−0.49,7.77]	24			
Body Fat Percentage	Intervention sequence	ST + ET	5	FE	0.31	[−1.45,2.08]	0	0.69	1	0.41
		ET + ST	1		2.00	[−1.58,5.58]	NA			
	Intervention Cycle	8W	2	FE	−0.23	[−3.02,2.57]	0	1.20	1	0.27
		12W	2		1.78	[−0.47,4.03]	0			
	Intervention frequency	Twice a week	3	FE	−0.33	[−2.80,2.13]	0	1.02	1	0.31
		3 times/week	2		1.33	[−0.74,3.39]	7			
1RM Lower Body Strength	Intervention sequence	ST + ET	5	RE	5.77	[−2.67,14.21]	95	0.12	1	0.73
		ET + ST	1		2.07	[−16.92,21.05]	74			
	Intervention interval	Interval	1	FE	4.58	[−26.48,35.65]	96	0.00	1	0.95
		Immediately	5		5.56	[−3.02,14.13]	95			
	Intervention Cycle	8W	2	FE	7.75	[−2.45,7.95]	90	0.07	1	0.79
		12W	2		10.55	[−6.78,27.87]	93			

BMI denotes Body Mass Index,CMJ denotes Vertical Jump Height,SLG denotes Standing Long Jump,FE stands for the fixed-effects model,RE stands for random effects model,ST + ET refers to strength training followed by endurance training,ET + ST refers to endurance training followed by strength training,"NA” indicates not applicable. The bolded sections indicate statistical significance.

#### 3.4.2 Reverse vertical jump

**Figure 4:**
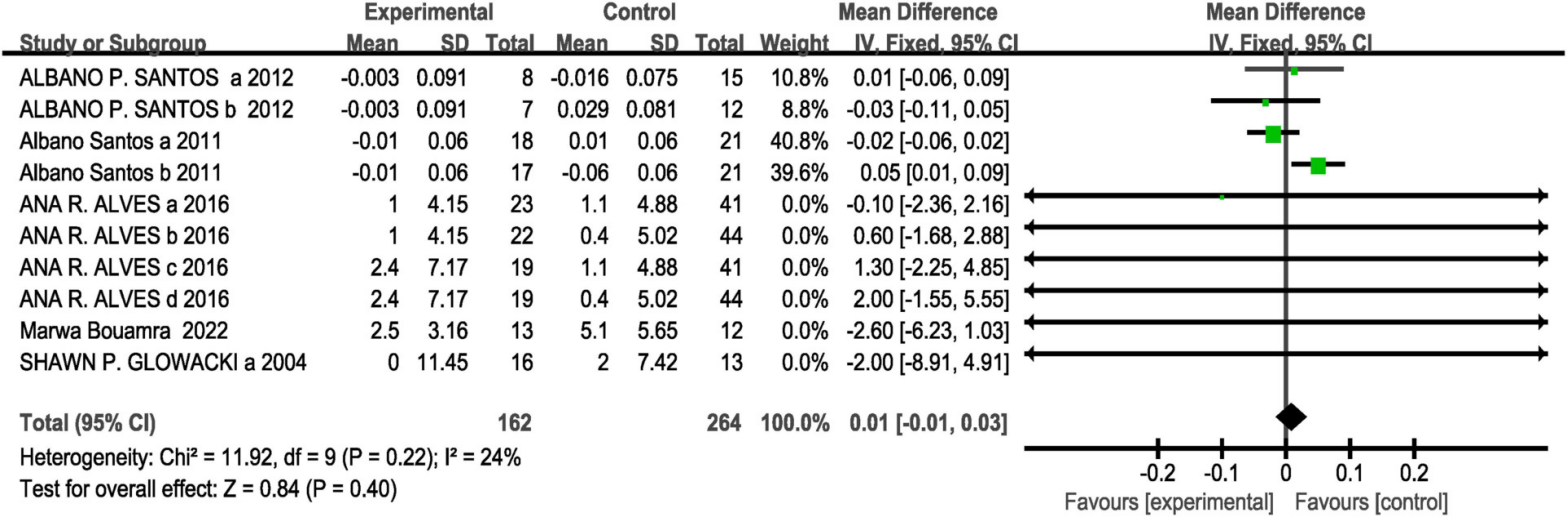
Effect of concurrent training on reverse vertical jump performance.

A total of five studies (10 research projects, 162 participants in the experimental group, and 264 in the control group) were included regarding the vertical jump test. As there was no heterogeneity in the results (I^2^= 24%, *P* > 0.1), a fixed-effects model was selected for analysis. The results showed no significant difference between the two groups [MD = 0.01, 95% CI: (−0.01, 0.03), *P* = 0.40 > 0.05], indicating that the effect of CT training on the backward vertical jump was not statistically significant compared to the control group, as shown in Figure 4.

Subgroup analysis results showed that for the interval [MD = 1.65, 95% CI: (−0.86, 4.16), I^2^ = 0%, *P* = 0.20 > 0.05], immediately [MD = 0.01, 95% CI: (−0.01, 0.03), I^2^ = 31%, *P* = 0.41 > 0.05], 8 weeks [MD = 0.01, 95% CI: (−0.01, 0.03), I^2^ = 27%, *P* = 0.40 > 0.05], 12 weeks [MD = −2.00, 95% CI: (−8.91, 4.91), *P* = 0.57 > 0.05], twice weekly [MD = 0.01, 95% CI: (−0.01, 0.03), I^2^ = 19%, *P* = 0.40 > 0.05], and 3 times/week [MD = 0.01, 95% CI: (−0.01, 0.03), *P* = 0.16 > 0.05] all showed no statistically significant differences in their effects on CMJ (*P* > 0.05 across subgroups) see Table 3. Since the CT training in the five included studies for this outcome measure all involved strength training followed by endurance training, no subgroup analysis was performed regarding the order of intervention.

#### 3.4.3 Standing long jump

**Figure 5:**
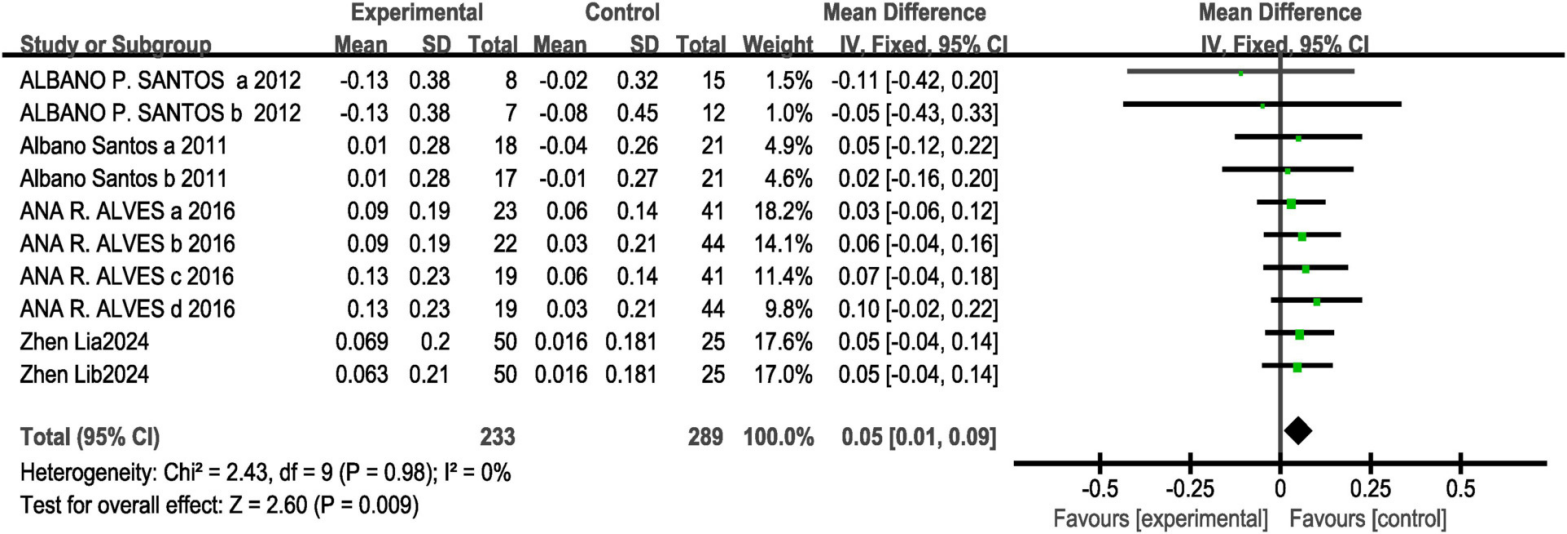
Forest plot showing the effect of concurrent training on the standing long jump.

A total of 4 studies (10 research projects, with 233 participants in the experimental group and 289 in the control group) were included regarding the standing long jump metric. As there was no heterogeneity in the results (I^2^ = 0%, *P* > 0.1), a fixed-effects model was selected for analysis. The results showed a statistically significant difference between the two groups [MD = 0.05, 95% CI: (0.01, 0.09), *P* = 0.009 < 0.05], which is statistically significant. This indicates that CT training can significantly improve standing long jump performance, as shown in Figure 5.

Subgroup analysis results showed that for the “delayed” group [MD = 0.07, 95% CI: (0.01, 0.13), I^2^ = 0%, *P* = 0.03 < 0.05] and the “immediate” group [MD = 0.04, 95% CI: (−0.01, 0.09), I^2^ = 0%, *P* = 0.11 > 0.05] indicate that a certain time interval between strength training and endurance training in CT is more beneficial for the standing long jump compared to performing endurance training immediately after strength training. The eight-week intervention period [MD = 0.05, 95% CI: (0.00, 0.10), I^2^ = 0%, *P* = 0.03 < 0.05] was statistically significant, whereas the 12-week intervention period [MD = 0.05, 95% CI: (−0.01, 0.11), I^2^ = 0%, *P* = 0.13 > 0.05] was not statistically significant see Table 3. Since the four included studies for this outcome measure all had experimental groups performing CT training with strength training followed by endurance training, and the intervention frequency was twice weekly for all, conditions for subgroup analysis were not met.

#### 3.4.4 Shot put (1-kg)

**Figure 6:**
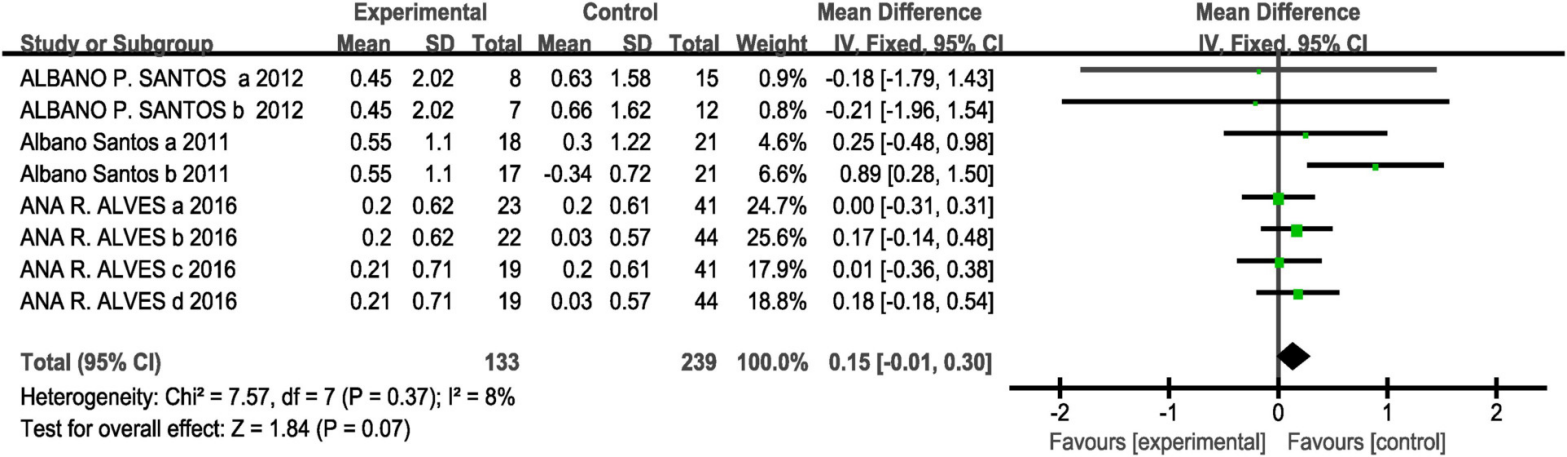
Forest plot showing the effect of concurrent training on medicine ball throw (1-kg).

Regarding the 1-kg medicine ball throw metric, three included studies (eight studies, 133 participants in the experimental group, and 239 in the control group) showed no heterogeneity (I^2^ = 8%, *P* > 0.1). Therefore, a fixed-effects model was selected for analysis. The results indicated no significant difference between the two groups [MD = 0.15, 95% CI: (−0.01, 0.30), *P* = 0.07 > 0.05], which is not statistically significant. This indicates that CT training is comparable to the other three training methods in enhancing upper-body strength and power in children and adolescents, as shown in Figure 6.

Subgroup analysis results showed that for interval training [MD = 0.10, 95% CI: (−0.16, 0.36), I^2^ = 0%, *P* = 0.46 > 0.05] and immediate [MD = 0.18, 95% CI: (−0.02, 0.37), I^2^ = 28%, *P* = 0.08 > 0.05] had no statistically significant difference in their effects on the 1-kg medicine ball throw (*P* > 0.05 between subgroups) see Table 3. Since the three included studies for this outcome measure all employed a CT training protocol consisting of strength training followed by endurance training, with an eight-week intervention period and twice-weekly training sessions, no subgroup analysis was performed for other intervention variables.

#### 3.4.5 Shot put (3 kg)

**Figure 7:**
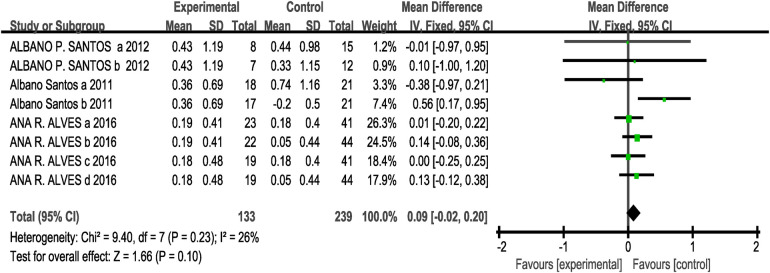
Forest plot showing the effect of concurrent training on medicine ball throw (3-kg).

Regarding this outcome measure, the three included studies (eight studies, 133 participants in the experimental group, and 239 in the control group) showed no heterogeneity (I^2^ = 26%, *P* > 0.1). Therefore, a fixed-effects model was selected for analysis. The results indicated no significant difference between the two groups [MD = 0.09, 95% CI: (−0.02, 0.20), *P* = 0.10 > 0.05], indicating that the effects of CT training on upper-body strength and power in children and adolescents were not statistically significant compared to the control group, as shown in Figure 7.

Subgroup analysis results showed that for interval [MD = 0.06, 95% CI: (−0.11, 0.24), I^2^ = 0%, *P* = 0.48 > 0.05] and immediate [MD = 0.10, 95% CI: (−0.03, 0.24), I^2^ = 43%, *P* = 0.12 > 0.05] had no statistically significant difference in their effects on the 3-kg medicine ball throw (*P* > 0.05 between subgroups) see Table 3. Since the three included studies for this outcome measure all employed a CT training protocol consisting of strength training followed by endurance training, with an eight-week intervention period and twice-weekly training sessions, no subgroup analysis was performed for other intervention variables.

#### 3.4.6 Sprint(20M)

**Figure 8:**
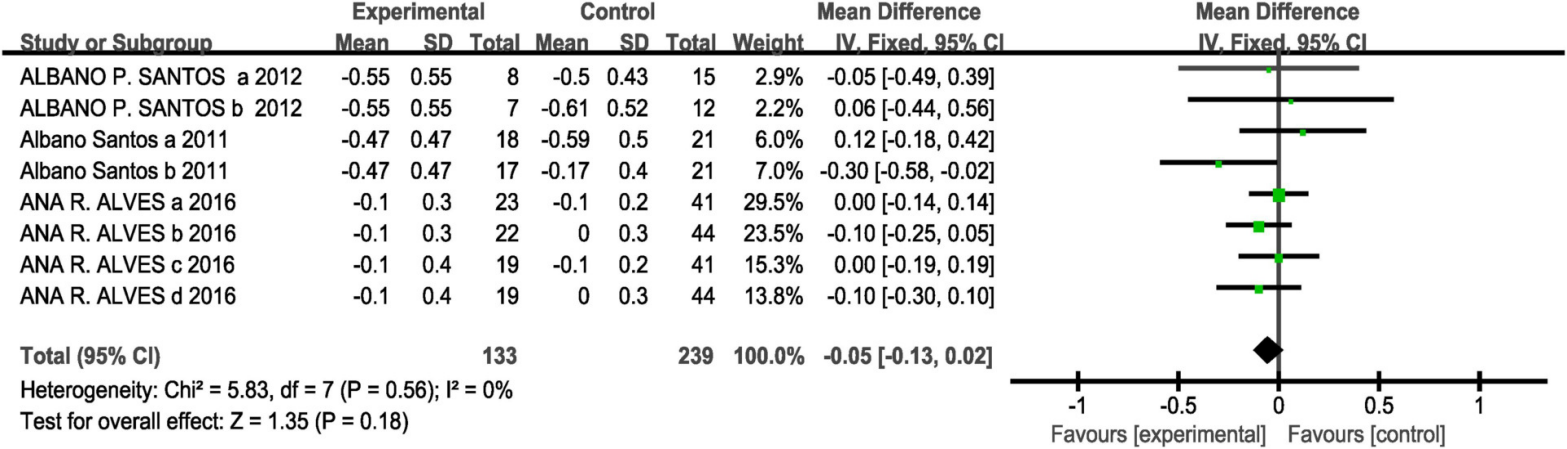
Forest plot showing the effect of concurrent training on the 20-meter sprint.

Regarding the 20-meter sprint performance metrics, a total of three studies (eight research projects, 133 participants in the experimental group, and 239 in the control group) were included. As there was no heterogeneity in the results (I^2^ = 0%, *P* > 0.1), a fixed-effects model was selected for analysis. The results showed no significant difference between the two groups [MD = −0.05, 95% CI: (−0.13, 0.02), *P* = 0.18 > 0.05], indicating no statistical significance. This suggests that the effect of CT training on explosive speed did not reach a significant level compared to other training methods, as shown in Figure 8.

Subgroup analysis results showed that interval training [MD = −0.05, 95% CI: (−0.19, 0.09), I^2^ = 0%, *P* = 0.50 > 0.05] and immediate [MD = −0.05, 95% CI: (−0.14, 0.04), I^2^ = 6%, *P* = 0.24 > 0.05] had no statistically significant effect on the 20-meter sprint (*P* > 0.05 between subgroups) see Table 3. Since the three included studies for this outcome measure all employed a training protocol in which the experimental groups performed strength training followed by endurance training, with an intervention period of eight weeks and two training sessions per week, the conditions for subgroup analysis were not met.

#### 3.4.7 VO_2_max

**Figure 9:**
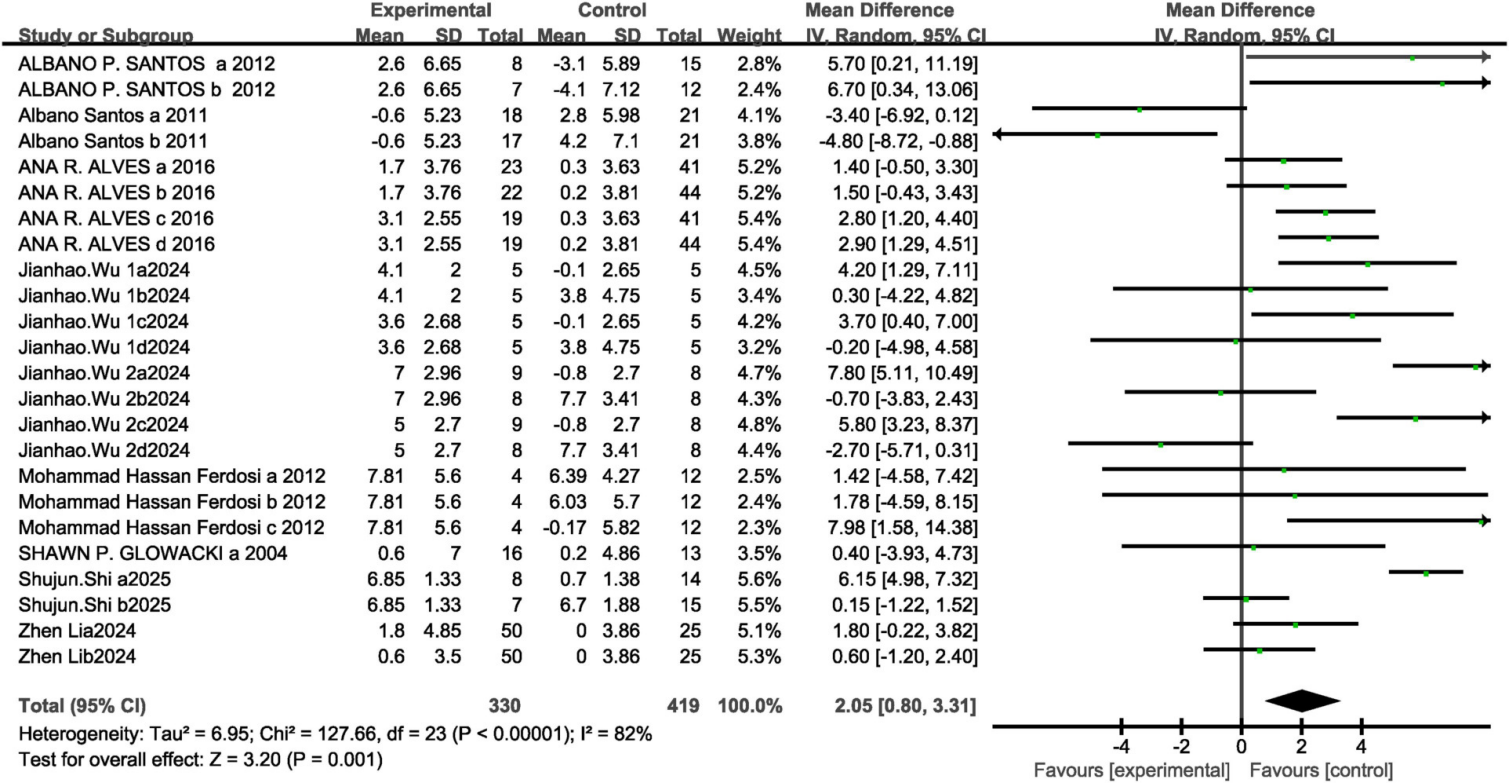
Forest plot of concurrent training effects on VO2max.

A total of nine studies (24 research projects, 330 participants in the experimental group and 419 in the control group) were included regarding the VO2max metric. The results showed high heterogeneity (I^2^= 82%, *P* < 0.1), so a random-effects model was selected for analysis. The analysis results revealed a significant difference between the two groups [MD = 2.05, 95% CI: (0.80, 3.31), *P* = 0.001 < 0.05] see Figure 9. Sensitivity analysis revealed that the estimated effect size and statistical significance of the VO2max metric remained stable when any single study was excluded, indicating robust meta-analytic results.

The subgroup analysis results indicate that, ST + ET（MD = 2.06, 95%CI: [0.73,3.39], I^2^ = 83%, *P* = 0.002>0.05), ET + ST (MD = 2.15, 95%CI: [−1.59,5.89], I^2^ = 42%, *P* = 0.26>0.05）CT training that begins with strength training followed by endurance training yields better effects on VO2max than training that starts with endurance training followed by strength training. Immediate [MD = 2.09, 95%CI: (0.50,3.68), I^2^ = 83%, *P* = 0.01 < 0.05], 8W [MD = 2.10, 95%CI: (0.61,3.59), I^2^ = 81%, *P* = 0.006 < 0.05], twice/week [MD = 1.93, 95%CI: (0.61,3.25), I^2^ = 84%, *P* = 0.004 < 0.05] The difference in the effect on VO₂max is statistically significant. and interval [MD = 1.99, 95%CI: (−0.11,4.09), I^2^ = 82%, *P* = 0.06 > 0.05], 12W [MD = 1.93, 95%CI: (−0.65,4.52), I^2^ = 85%, *P* = 0.14 > 0.05], 3 times/week [MD = 3.64, 95%CI: (−0.49,7.77), I^2^ = 24%, *P* = 0.08 > 0.05] None were statistically significant see Table 3.

#### 3.4.8 Body fat percentage

**Figure 10:**
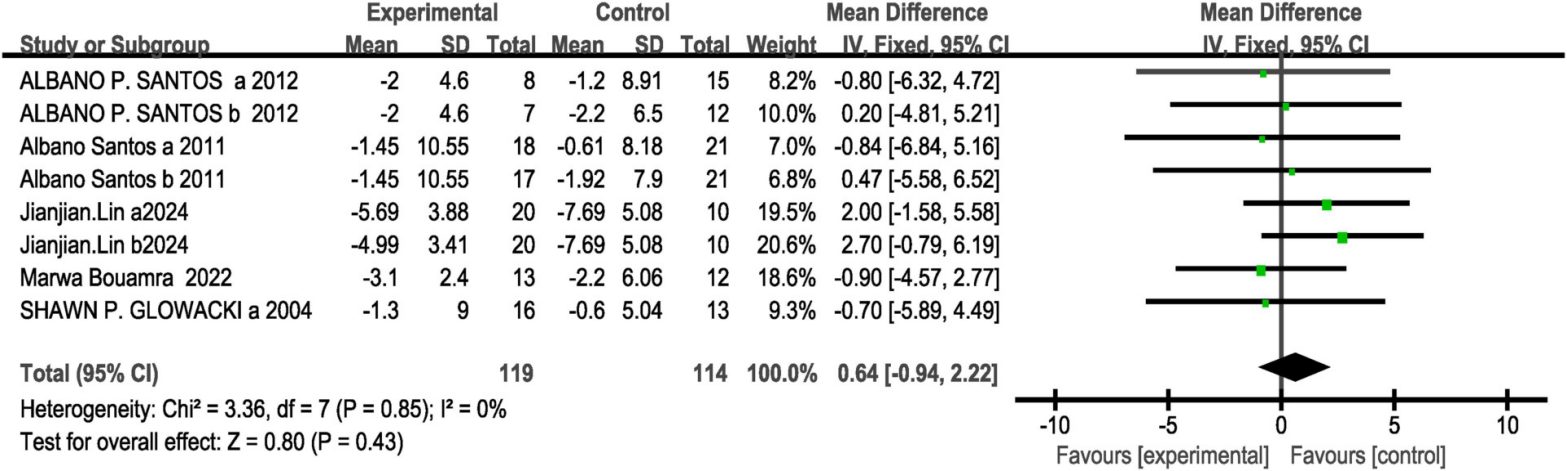
Forest plot of the effect of concurrent training on body Fat percentage.

Regarding the outcome measure of body fat percentage, a total of five studies were included (eight research projects, 119 participants in the experimental group and 114 in the control group). The results showed no heterogeneity (I^2^= 0%, *P* > 0.1), thus a fixed-effects model was selected for analysis. The analysis revealed no significant difference between the two groups [MD = 0.64, 95% CI: (−0.94, 2.22), *P* = 0.43 > 0.05]. No statistically significant difference was observed, indicating that CT training did not produce a significantly greater effect on body mass compared to other training methods, as shown in Figure 10.

The subgroup analysis results indicate that, ST + ET (MD = 0.31, 95%CI: [−1.45,2.08], I^2^ = 0%, *P* = 0.73>0.05), ET + ST (MD = 2.00, 95%CI: [−1.58,5.58], *P* = 0.27>0.05), 8W (MD = −0.23, 95%CI: [−3.02,2.57], I^2^ = 0%, *P* = 0.87>0.05), 12W (MD = 1.78, 95%CI: [−0.47,4.03], I^2^ = 0%, *P* = 0.12>0.05), twice/week (MD = −0.33, 95%CI: [−2.80,2.13], I^2^ = 0%, *P* = 0.79>0.05), 3 times/week (MD = 1.33, 95%CI: [−0.74,3.39], I^2^ = 7%, *P* = 0.21>0.05) The differences in effects on body fat percentage were not statistically significant (*P* > 0.05 between subgroups) see Table 3. Since all five studies included in this outcome measure conducted CT training without a time interval between strength training and endurance training, no subgroup analysis was performed for intervention intervals.

#### 3.4.9 Maximum lower-body strength

**Figure 11:**
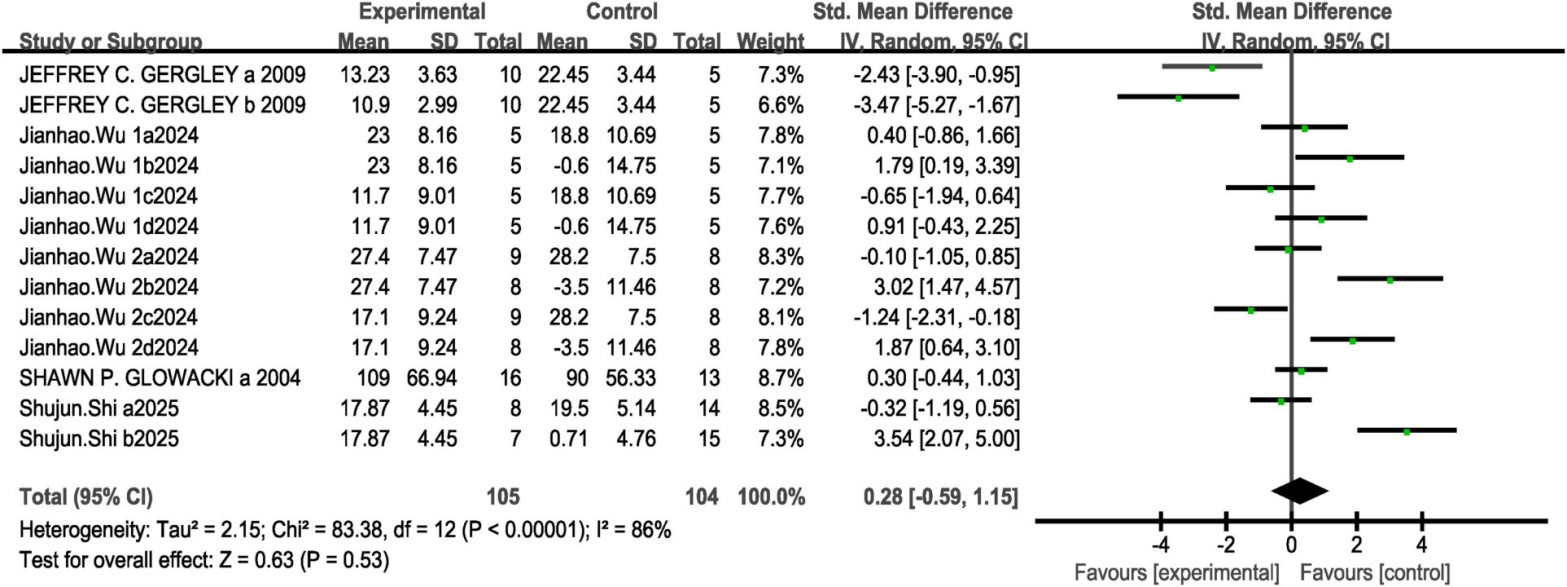
Forest plot of the effect of concurrent training on 1RM lower-body strength.

Regarding 1RM lower-body strength indicators, a total of five studies were included (13 research projects, 105 participants in the experimental group and 104 in the control group). The results showed high heterogeneity (I^2^ = 86%, *P* < 0.1), so a random-effects model was selected for analysis. The analysis revealed no significant difference between the two groups [MD = 0.28, 95% CI: (−0.59, 1.15), *P* = 0.53 > 0.05], as shown in Figure 11. Sensitivity analysis revealed that the estimated effect size and statistical significance of the 1RM lower-body strength metric remained stable when any single study was excluded, indicating robust meta-analytic results.

The subgroup analysis results indicate that, ST + ET (MD = 5.77, 95%CI: [−2.67,14.21], I^2^ = 95%, *P* = 0.18>0.05）, ET + ST（MD = 2.07, 95%CI: [−16.92,21.05], I^2^ = 74%, *P* = 0.83>0.05）, interval（MD = 4.58, 95%CI: [−26.48,35.65], I^2^ = 96%, *P* = 0.77>0.05）, Immediate（MD = 5.56, 95%CI: [−3.02,14.13], I^2^ = 95%, *P* = 0.20>0.05）, 8W（MD = 7.75, 95%CI: [−2.45,17.95], I^2^ = 90%, *P* = 0.14>0.05）, 12W（MD = 10.55, 95%CI: [−6.78,27.87], I^2^ = 93%, *P* = 0.23>0.05）The differences in the effects on 1RM maximum lower-limb strength were not statistically significant (*P* > 0.05 between subgroups) see Table 3. Since all five studies included in this outcome measure conducted CT training twice weekly, no subgroup analysis was performed based on intervention frequency.

### 3.5 Publication bias

**Figure 12:**
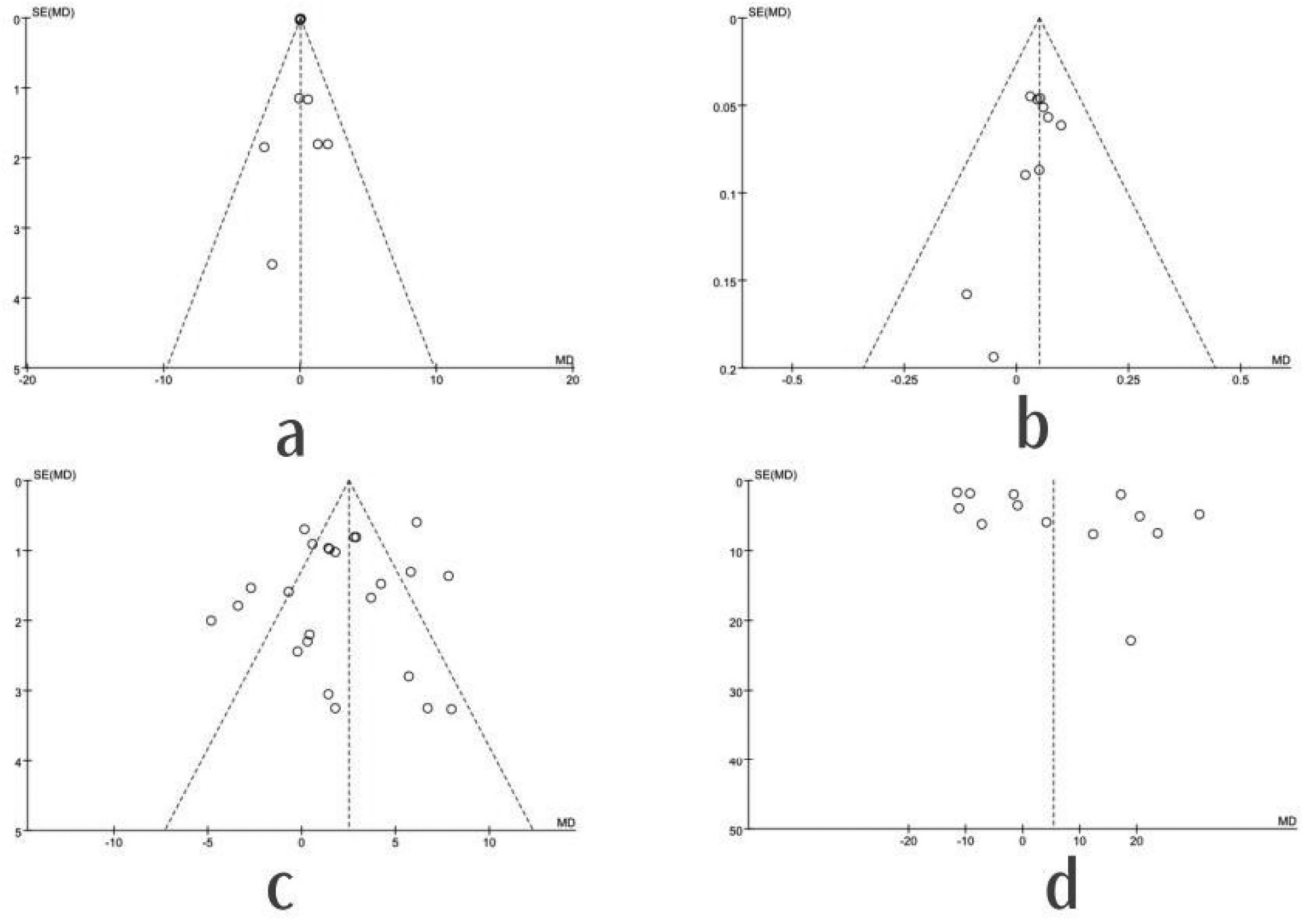
Risk of bias plot for each outcome indicator. **a**: Backward vertical jump publication bias plot; **b**: Standing long jump publication bias plot; **c**: VO2max publication bias plot; **d**: 1RM lower-body strength publication bias plot.

Figure 12 presents a funnel plot constructed from outcomes included in ten or more studies. The scatter plots for each outcome measure exhibit relatively dispersed distributions that were not completely symmetrical on both sides, indicating the potential presence of publication bias. Subsequently, Egger's linear regression test was applied. In this study, the results for the reverse vertical jump (t = 0.02, *P* = 1.000 > 0.05), standing long jump (t = −0.20, *P* = 0.484 > 0.05), VO₂max (t = 0.09, *P* = 0.539 > 0.05), and 1RM lower-body strength (t = 0.25, *P* = 0.251 > 0.05) Egger's correlation test revealed that in this study, BMI (z = 0.10, *P* = 0.916 > 0.05), 1-kg medicine ball (z = 0.45, *P* = 0.652 > 0.05), 3-kg medicine ball (z = −0.07, *P* = 0.939 > 0.05), 20-m sprint (z = −0.03, *P* = 0.973 > 0.05), and body fat percentage (z = −1.02, *P* = 0.304 > 0.05) showed no significant publication bias, indicating overall high quality.

## 4 Discussion

CT is applied to sports that require both muscular strength and a certain level of cardiorespiratory endurance, such as cross-country skiing and basketball (38–40).Strong muscle strength and endurance are key determinants of success in many sports (41, 42). Numerous studies have demonstrated that CT significantly promotes reductions in the prevalence of fatty liver disease, total cholesterol (TC), and low-density lipoprotein cholesterol (LDL-c) among obese adolescents (43–46). Currently, most scholars have conducted limited research on CT exposure. Therefore, it is essential to conduct a comprehensive evaluation of CT's impact on children and adolescents and perform quantitative analysis. This study employed systematic review and meta-analysis methods to comprehensively evaluate the quantitative effects of concurrent training (CT) vs. single-mode training (strength training ST, endurance training ET) and no training (GT) on body mass, upper and lower limb muscle strength, explosive power, and cardiorespiratory endurance in children and adolescents. The study results indicate that compared to single-mode training (strength training ST, endurance training ET) and no training (GT), Concurrent training demonstrates significant advantages in enhancing cardiorespiratory endurance (VO2max) and lower-body explosive power (standing long jump). No significant differences were observed in metrics such as BMI, body fat percentage, upper-body strength, and 20-meter sprint performance. This finding provides new evidence-based support for physical health interventions in children and adolescents, offering valuable reference for designing school physical education curricula and developing youth training programs.

This study did not observe an interference effect in the pediatric and adolescent population, meaning concurrent training did not inhibit strength development but instead demonstrated synergistic enhancement effects in certain indicators (47, 48). This study supports the “interference hypothesis” proposed by Kraemer et al. in 1995 (49), and consistent with the findings of numerous studies on children and adolescents in recent years (24). Training frequency is a key factor contributing to the incompatibility between ST and ET (50, 51),Hickson (19) the study involved training 11 times per week, leading to “overtraining,” which may be one reason for the incompatibility. Murach (50) training with a frequency of 2 days per week (or up to 3 days) maximizes muscle hypertrophy adaptation. Multiple studies support this view: training more than three times per week produces an interference effect (49), Interference level reduced to ≤3 training sessions (22, 41, 52). The rational arrangement of training intervention variables such as intensity, frequency, sequence, and interval timing helps mitigate interference effects. All 30 studies included in this review employed a training regimen of 2 or 3 sessions per week at moderate intensity, which explains why CT did not induce an “interference effect.”

Based on outcome measures such as BMI and body fat percentage, CT showed no significant differences in body composition among children and adolescents across various exercise regimens. Although physical exercise is an effective method for promoting metabolic reactions and improving fat accumulation, the rate of body fat metabolism is related to the amount of exercise (53), However, since children and adolescents are in a period of rapid physical development, coupled with the continuous improvement in quality of life, adequate nutrient intake among participants may lead to an energy surplus (27). However, the exercise interventions in this study were all of moderate intensity, insufficient to expend excess energy, resulting in no significant differences in body mass outcome measures. Although the outcome measures in this study—including 1RM lower-body strength and CMJ—did not reach statistical significance, the training results indicate that all measures showed a certain degree of improvement. This is consistent with the findings of CHTARA et al. (54). In this study, all three training groups ST, ST + ET, and ET + ST exhibited increases in 1RM lower-body strength. The study indicates that ST, ST + ET, and ET + ST training groups all enhance muscle strength capacity. Furthermore, the increase in 1RM lower-body strength was comparable between the ST and ST + ET groups. This result is consistent with the findings of Glowacki et al. (35).

Based on the SLJ outcome measures in this study, Concurrent training significantly enhances lower-body explosive power in children and adolescents [MD = 0.05, 95% CI: (0.01, 0.09), *P* < 0.05]. Subgroup analysis further indicates that incorporating intervals between strength and endurance training during CT training is more conducive to developing lower-body explosive power. This is consistent with the findings of Alves et al. (31). Some research findings suggest that CT training may interfere with muscle strength development (34, 55, 56), As a result, there were no significant differences in outcome measures such as CMJ, medicine ball throw (1-kg, 3-kg), and lower-body strength in this study. It may be closely related to acute fatigue and neuromuscular adaptations distinct from those seen in endurance or strength training (57). Additionally, Sale et al (56). found that, In CT training, the lack of a time interval between strength and endurance training may inhibit strength development but does not inhibit VO2max.

Regarding the outcome measure for the 20-meter sprint in this study, the analysis yielded the following results: [MD = −0.05, 95% CI: (−0.13, 0.02), *P* > 0.05]. The results were not statistically significant, and corresponding subgroup analyses also failed to reveal any significant differences. However, the running speed of each training group showed a corresponding improvement. This is consistent with the findings of Maro et al. (58). These findings indicate that when it comes to improving running speed in children and adolescents, additional endurance training does not yield greater benefits than strength training. Another contributing factor is that while the physical activities children and adolescents engage in during physical education classes are generally low-intensity, certain sports—such as basketball and soccer—involve periods of high-intensity performance interspersed with low-intensity phases. These alternating phases may be a reason for improved running speed (34).

This study found that CT significantly enhances VO₂max [MD = 2.05, 95% CI: (0.80, 3.31), *P* < 0.05]. Subgroup analysis further indicated that the training sequence prioritizing strength over endurance, the arrangement of non-interval training, a training frequency of twice weekly, and an 8-week intervention period yielded superior outcomes. CT can enhance VO2max in children and adolescents (59), This effect was more pronounced in individuals aged 18 years and older, with no decline in aerobic capacity observed. Consistent with the findings of Sanchez-Moreno et al (48).and Li Gaofeng et al. (60), Consistent with the physical development model for children and adolescents established by Lloyd et al. (61), enhanced metabolic capacity around puberty, coupled with the integration of central and peripheral cardiovascular systems and neuromuscular function, will significantly improve endurance capacity.

Subgroup analysis of VO2max outcomes revealed that combining strength and endurance training within the same timeframe during CT training effectively enhances aerobic capacity. This is inconsistent with the findings of Robineau et al (62), The conclusion drawn is that concurrent training during the same time period does not yield optimal improvements in aerobic capacity, but better adaptive changes can be observed when training is spaced apart. The reason for this difference may be related to the intensity of endurance training. Research by Sousa et al. (63) indicates that during CT training, Choosing moderate-intensity endurance training can better improve aerobic capacity. Santos et al. (34) concluded that, Among children and adolescents, aerobic capacity can only be effectively enhanced when both strength and endurance training are conducted simultaneously within the classroom setting. The primary reason for this phenomenon is that combining strength and endurance training in a single session more effectively enhances vascularization and oxidative enzyme activity [such as capillary density and area, as well as succinate dehydrogenase (SDH) activity] (64). However, regarding the SLJ outcome measures in this study, unlike the VO2max outcome measures, the time interval does affect the efficacy of CT on SLJ (62, 65), Robineau et al. (62) compared the effects of 0-hour, 6-hour, and 24-hour rest intervals on rugby players, finding that the 24-hour interval yielded the greatest strength gains.

This paper also has certain limitations, which will be addressed in future research:1. This study employed stringent inclusion and exclusion criteria, resulting in a limited number of participants.2. Some literature fails to specify strength characteristics and employs inconsistent metrics, thereby precluding analysis of strength indicators.3. VO₂max is influenced by multiple factors, and there have been few CT-related studies on different age groups. Future research in this area could be expanded.

## 5 Conclusion

In summary, when conducting concurrent training for children and adolescents, limit training sessions to ≤3 times per week to avoid interference effects. CT training significantly enhances lower-body explosive power and aerobic capacity in children and adolescents. The most effective approach involves prioritizing strength training over endurance training, with no interval between the two sessions. It is also recommended that an intervention period of 8 weeks will yield more significant effects on training outcomes. Additionally, this study found no significant differences in other outcome measures. Given the influence of exercise intensity, the above conclusions require further validation and investigation.

## Data Availability

The original contributions presented in the study are included in the article/Supplementary Material, further inquiries can be directed to the corresponding author/s.
